# Endocytosis and lysosomal degradation of GluA2/3 AMPARs in response to oxygen/glucose deprivation in hippocampal but not cortical neurons

**DOI:** 10.1038/s41598-017-12534-w

**Published:** 2017-09-26

**Authors:** Zsombor Koszegi, Maria Fiuza, Jonathan G. Hanley

**Affiliations:** Centre for Synaptic Plasticity and School of Biochemistry, Biomedical Sciences Building, University of Bristol, University Walk, Bristol, BS8 1TD UK

## Abstract

Global cerebral ischemia results in oxygen and glucose deprivation (OGD) and consequent delayed cell death of vulnerable neurons, with hippocampal CA1 neurons more vulnerable than cortical neurons. Most AMPA receptors (AMPARs) are heteromeric complexes of subunits GluA1/GluA2 or GluA2/GluA3, and the presence of GluA2 renders AMPARs Ca^2+^-impermeable. In hippocampal CA1 neurons, OGD causes the synaptic expression of GluA2-lacking Ca^2+^-permeable AMPARs, contributing to toxic Ca^2+^ influx. The loss of synaptic GluA2 is caused by rapid trafficking of GluA2-containing AMPARs from the cell surface, followed by a delayed reduction in GluA2 mRNA expression. We show here that OGD causes endocytosis, lysosomal targeting and consequent degradation of GluA2- and GluA3-containing AMPARs, and that PICK1 is required for both OGD-induced GluA2 endocytosis and lysosomal sorting. Our results further suggest that GluA1-containing AMPARs resist OGD-induced endocytosis. OGD does not cause GluA2 endocytosis in cortical neurons, and we show that PICK1 binding to the endocytic adaptor AP2 is enhanced by OGD in hippocampal, but not cortical neurons. We propose that endocytosis of GluA2/3, caused by a hippocampal-specific increase in PICK1-AP2 interactions, followed by PICK1-dependent lysosomal targeting, are critical events in determining changes in AMPAR subunit composition in the response to ischaemia.

## Introduction

AMPARs mediate the majority of fast synaptic excitation in the brain, and the precise regulation of AMPA receptor (AMPAR) trafficking is crucial to excitatory neurotransmission, synaptic plasticity and the consequent formation and modification of appropriate neural circuits during learning and memory^1–3^. AMPARs are tetrameric assemblies of subunits GluA1-4, and the vast majority are heteromers containing GluA1/GluA2, or GluA2/GluA3, with GluA1/GluA2 complexes thought to be the most common^4–6^. The presence of GluA2 is critical, because it renders AMPARs Ca^2+^ impermeable, hence maintaining an appropriately low cytoplasmic Ca^2+^ concentration during basal synaptic transmission^7^. However, a small population of GluA2-lacking, Ca^2+^-permeable (CP-)AMPARs exists, which may be GluA1 homomers or GluA1/GluA3 heteromers. The majority of synapses, especially on pyramidal neurons, do not express GluA2-lacking AMPARs under resting conditions, and precise regulation of their synaptic expression is important for Ca^2+^ signalling events, for example during Long-Term Potentiation (LTP) expression^8^. However, dysregulation of these processes can lead to a prolonged synaptic incorporation of CP-AMPARs, resulting in excessive Ca^2+^ influx, which causes synaptic dysfunction and cell death (excitotoxicity) in a number of diseases including brain ischemia, traumatic brain injury and chronic disorders such as Huntington’s disease^9^. Therefore, increased knowledge of the subunit-specific mechanisms of AMPAR trafficking is critical to our understanding of these disease states.

Brain ischemia occurs when the blood supply to the brain is interrupted, for example by occlusion following a stroke, or as a result of cardiac arrest. The OGD that occurs during ischemia exposes neurons to metabolic stress, which causes widespread depolarization of the neuronal plasma membrane, massive release of the excitatory neurotransmitter glutamate and overexcitation of ionotropic glutamate receptors, which causes a sustained elevation of intracellular Ca^2+^, and consequently a delayed, selective cell death^10^. The main pathway by which excitotoxicity is initiated is Ca^2+^ influx through NMDARs, which triggers a number of signalling pathways, resulting in numerous downstream effects^11,12^. In hippocampal CA1 neurons these include changes in synaptic AMPAR subunit composition resulting in the expression of GluA2-lacking CP-AMPARs. This leads to Ca^2+^ influx that contributes to delayed cell death hours to days later^13^. Two distinct phases to this process have been described; an initial rapid trafficking phase involving an NMDAR-dependent removal of GluA2 subunit from the plasma membrane^14–16^, and a later phase in which GluA2 subunit mRNA expression and consequently protein levels are reduced^17,18^.

Following global cerebral ischemia, specific regions of the brain show greater neuronal injury than others, suggesting different mechanisms are recruited in response to insult. Pyramidal neurons in the CA1 hippocampal region are the most vulnerable, while their CA3 counterparts are resistant^19^. Although cortical pyramidal neurons are affected by ischemia, they are less vulnerable than those in hippocampal CA1 following a global insult^20^, suggesting that different cell-type specific mechanisms are activated in response to OGD. We previously demonstrated that OGD causes a loss of surface-expressed GluA2 in hippocampal neurons, but not in cortical neurons^16^. However, the molecular mechanisms that underlie this difference are unknown.

The AMPAR trafficking events caused by OGD share some common features with those that underlie the expression of LTP and Long-Term Depression (LTD), which are forms of synaptic plasticity thought to be the cellular correlates of learning and memory. In response to LTD induction, internalized AMPARs are targeted for lysosomal degradation instead of recycling back to the plasma membrane, hence providing a mechanism for the long-term removal of synaptic receptors^21,22^. The fate of GluA2-containing AMPARs internalized in response to OGD is unknown, and is likely to be a critical factor in the long-term consequences of brain ischaemia. In this study, we therefore asked whether OGD causes lysosomal targeting of GluA2-containing AMPARs. Moreover, while we previously demonstrated that PICK1 is involved in the OGD-induced loss of surface GluA2^15^, the precise trafficking events that are regulated by PICK1 are unclear. Since it has been suggested that PICK1 is involved in both endocytosis and endosomal sorting of AMPARs in response to NMDAR activation in neurons (refs^23–28^), we investigated a role for PICK1 in one or both of these trafficking events. We show that OGD causes a selective endocytosis of endogenous GluA2/A3 receptors from the plasma membrane, which are subsequently trafficked to lysosomes for degradation. Lysosomal sorting and degradation were blocked by acute application of a cell-permeant peptide that disrupts PICK1-GluA2 interactions. We observed these trafficking events in hippocampal neurons, but not cortical neurons, which can be explained by an OGD-induced increase in PICK1-AP2 binding to drive AMPAR endocytosis specifically in hippocampal neurons.

## Results

### Internalized GluA2 is degraded in lysosomes following OGD in hippocampal neurons

**Figure 1:**
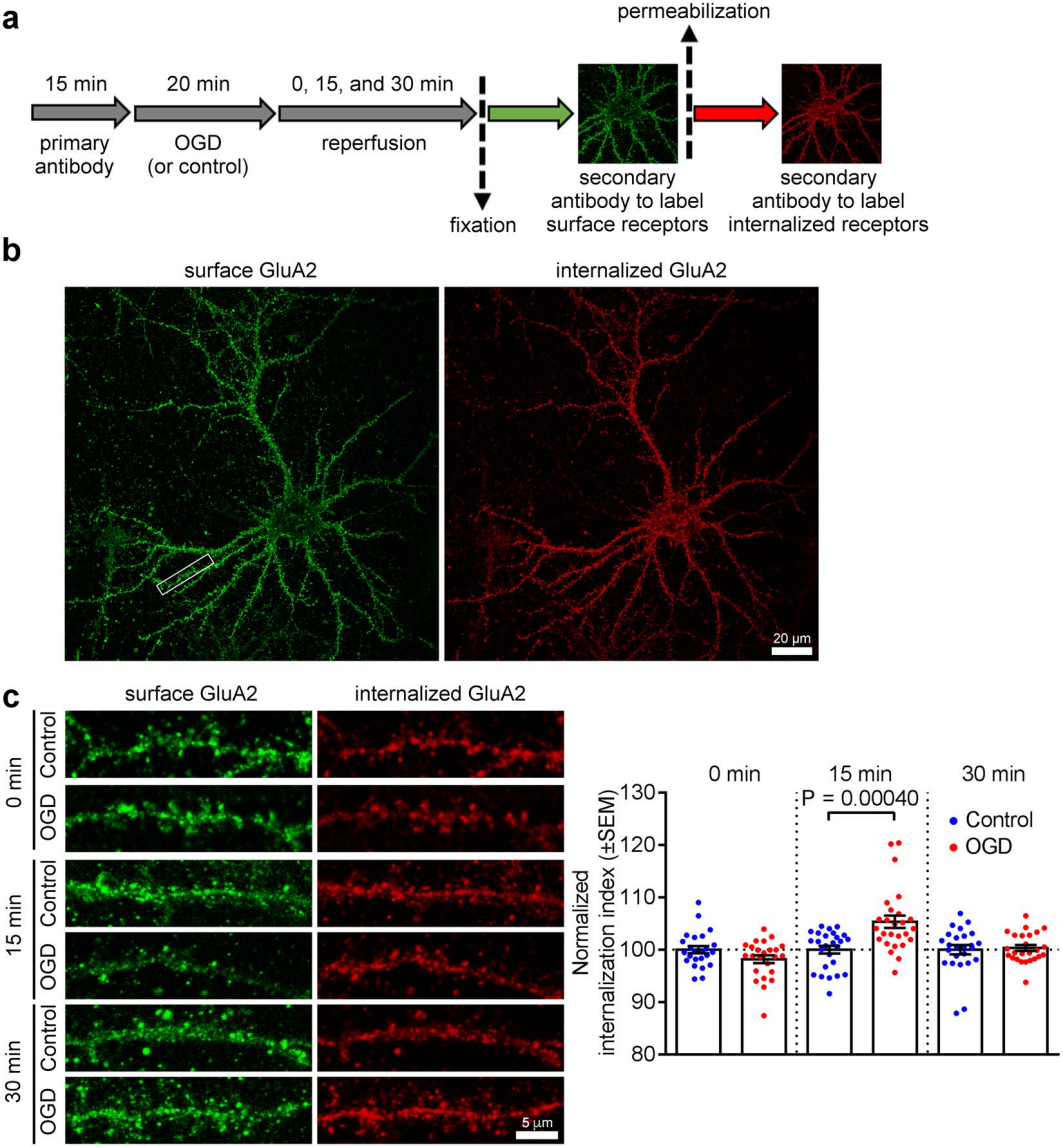
Endocytosis of endogenous GluA2 in response to OGD/reperfusion in hippocampal neurons. (**a**) Schematic of antibody-feeding protocol. Neurons were live labeled with anti-GluA2 primary antibodies for 15 min followed by 20 min of OGD and 0, 15, or 30 min of reperfusion. Neurons were then fixed briefly with PFA and green secondary antibodies were used to label the remaining surface receptors. After permeabilization, red secondary antibodies were used to label internalized receptors. (**b**) Confocal images of a cultured hippocampal neuron live-labeled with anti-GluA2 antibodies prior to 20 min OGD and 15 min reperfusion. The intensities of the remaining surface (green) and internalized (red) endogenous GluA2 were measured and used to calculate the internalization index (internalized/internalized + surface). Three secondary dendrites (white rectangle) with an average length of 20 to 30 µm were used from each neuron for intensity quantifications. (**c**) Representative confocal images of dendrites from neurons treated as in (**a**) Graph indicates a significant increase in GluA2 internalization following OGD and 15 min reperfusion. Two-tailed Student’s t-test.

**Figure 2:**
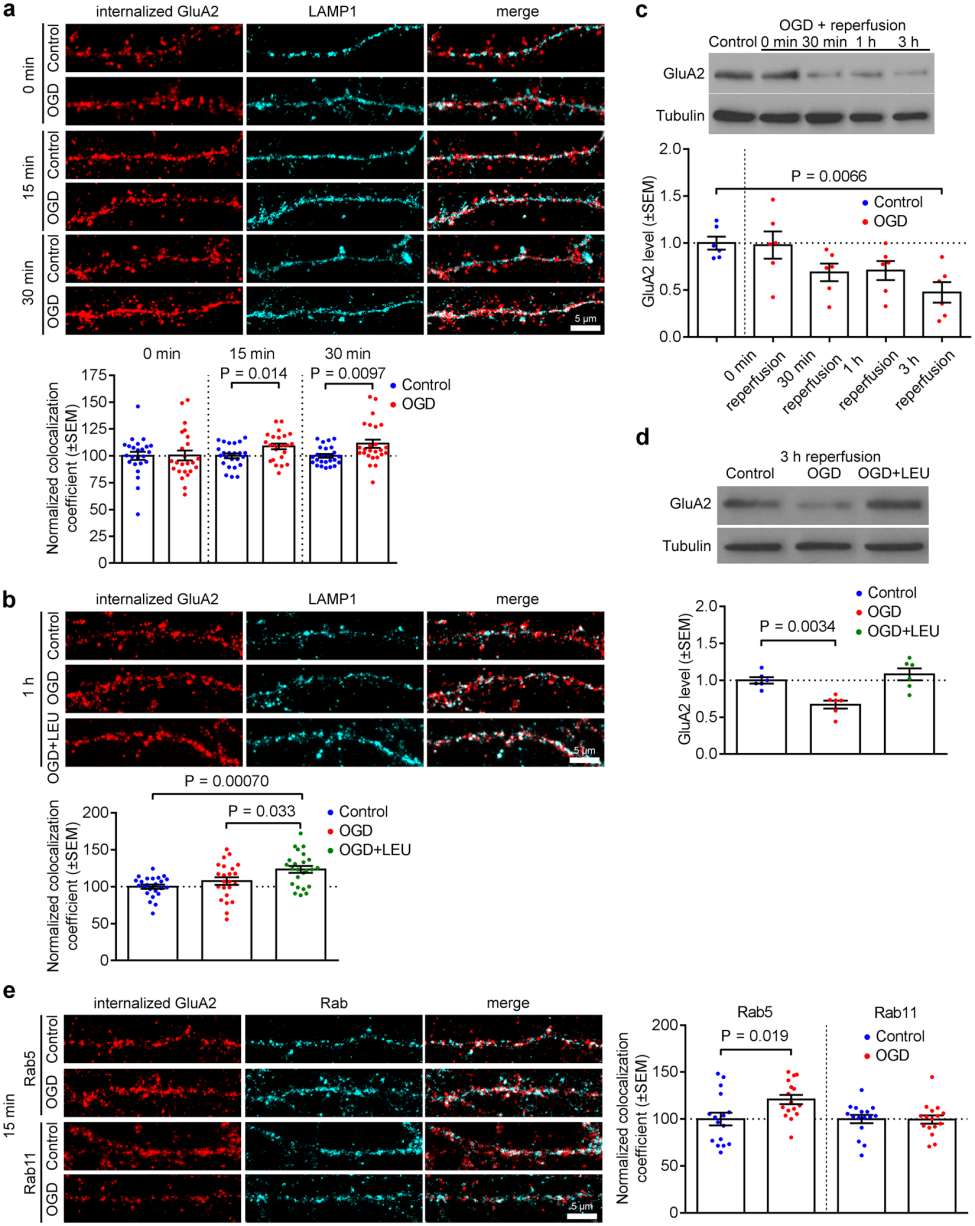
Lysosomal targeting of internalized endogenous GluA2 following OGD in hippocampal neurons. (**a**) OGD/reperfusion causes the targeting of internalized endogenous GluA2 to lysosomes in dendrites of cultured hippocampal neurons. Neurons were live-labeled with anti-GluA2 antibodies prior to 20 min OGD and 0, 15, 30 min reperfusion. After fixation, internalized GluA2 was labeled with red secondary antibodies, and neurons were also immunostained for the lysosomal marker LAMP1 (cyan). Representative confocal images are shown. Pearson’s coefficient of colocalization between internalized GluA2 and the lysosomal marker LAMP1 was determined. Graph indicates a significant increase after 15 and 30 min reperfusion. Two-tailed Student’s t-test. (**b**) Inhibition of lysosomal degradation prolongs localization of endogenous GluA2 in lysosomes. The application of leupeptin (100 μg/ml for 3 h before and during OGD/reperfusion) significantly increased the colocalization between internalized GluA2 and LAMP1 after 1 h of reperfusion. Representative confocal images are shown. One-way ANOVA followed by Tukey’s test. (**c**) Endogenous GluA2 is degraded following OGD/reperfusion in cultured hippocampal neurons. Cell lysates were analyzed by Western blotting. Graph shows total GluA2 levels following 20 min OGD and 0, 30 min, 1 h and 3 h reperfusion. One-way ANOVA followed by Dunnett’s test. Blots shown are cropped; full-length blots are shown in Supplementary Fig. S3. (**d**) GluA2 degradation is blocked by the lysosomal inhibitor leupeptin. Leupeptin was applied as in b, above, and neurons exposed to OGD followed by 3 h reperfusion. Cell lysates were analyzed by Western blotting. Graph shows total GluA2 levels. One-way ANOVA followed by Dunnett’s test. Blots shown are cropped; full-length blots are shown in Supplementary Fig. S3. (**e**) OGD/reperfusion causes the targeting of internalized endogenous GluA2 to early endosomes but not recycling endosomes in dendrites of cultured hippocampal neurons. Neurons were live-labeled with anti-GluA2 antibodies prior to 20 min OGD and 15 min reperfusion. After fixation, internalized GluA2 was labeled with red secondary antibodies, and neurons were also immunostained for the early endosome marker Rab5 or the recycling endosome marker Rab11 (cyan). Representative confocal images are shown. Pearson’s coefficients of colocalization between internalized GluA2 and Rab5 or Rab11 were determined. Graph indicates a significant increase for Rab5, but not Rab11. Two-tailed Student’s t-tests.

To investigate the fate of internalized GluA2-containing AMPARs after OGD in hippocampal neurons, we initially demonstrated a significant OGD-induced increase in the endocytosis of endogenous GluA2-containing AMPARs using an antibody-feeding assay (Fig. 1a,b) quantifying the proportion of endogenous GluA2 internalized from the cell surface. In these assays, only receptors that originated on the cell surface are labelled with antibody, and have therefore trafficked through the endosomal system before recycling or sorting to lysosomes at later time points. OGD caused a significant increase in GluA2 internalization index, calculated as internalized GluA2/(surface + internalized GluA2) (Fig. 1c). We analysed the targeting of endocytosed GluA2 to lysosomes by measuring the colocalization between the lysosomal marker LAMP1 and internalized GluA2 (iGluA2). While there was no change in iGluA2/LAMP1 colocalization immediately after OGD compared to controls, by 15 min post-OGD we observed a small but significant increase, which persisted up to 30 min (Fig. 2a). Interestingly, this was specific to dendrites, and no change in iGluA2/LAMP1 colocalization was seen in neuronal cell bodies (Supplementary Fig. S1). To investigate this further, we carried out the same analysis at 1 h after OGD, and found that iGluA2/LAMP1 colocalization was indistinguishable from controls, suggesting that GluA2 had been subjected to lysosomal degradation at this later time point (Fig. 2b). To test this hypothesis directly, we treated cultures with leupeptin, which inhibits lysosomal degradation, for 3 h before and throughout OGD/reperfusion^22^. Leupeptin restored the increase in iGluA2/LAMP1 colocalization at 1 h after OGD (Fig. 2b), supporting the hypothesis that OGD causes degradation of GluA2 in lysosomes by 1 h after OGD. The loss of surface GluA2 caused by OGD was unaffected by leupeptin treatment, indicating that blockade of lysosomal degradation does not cause the recycling of GluA2 to the surface (Supplementary Fig. S2). Consistent with these observations, Western blotting demonstrated that OGD caused a gradual decrease in total GluA2 levels in hippocampal neurons, which reached statistical significance at 3 h after OGD (Fig. 2c). The OGD-induced loss of total GluA2 was completely blocked by leupeptin, confirming lysosomal degradation (Fig. 2d). To explore further the endosomal sorting of AMPARs after OGD, we analysed colocalization between internalized GluA2 and Rab5 or Rab11, which are markers for early and late endosomes, respectively. Colocalization with Rab5 was significantly increased by OGD at 15 min after insult, whereas colocalization with Rab11 was unaffected, suggesting that OGD causes GluA2-containing AMPARs to traffic through early endosomes, but not recycling compartments (Fig. 2e). These results indicate that OGD causes an increase in endosomal sorting of endogenous GluA2-containing AMPARs to lysosomes (via early endosomes), where a proportion of GluA2 is degraded.

### Dual role for PICK1 in endocytosis and lysosomal targeting of GluA2 in response to OGD

**Figure 3:**
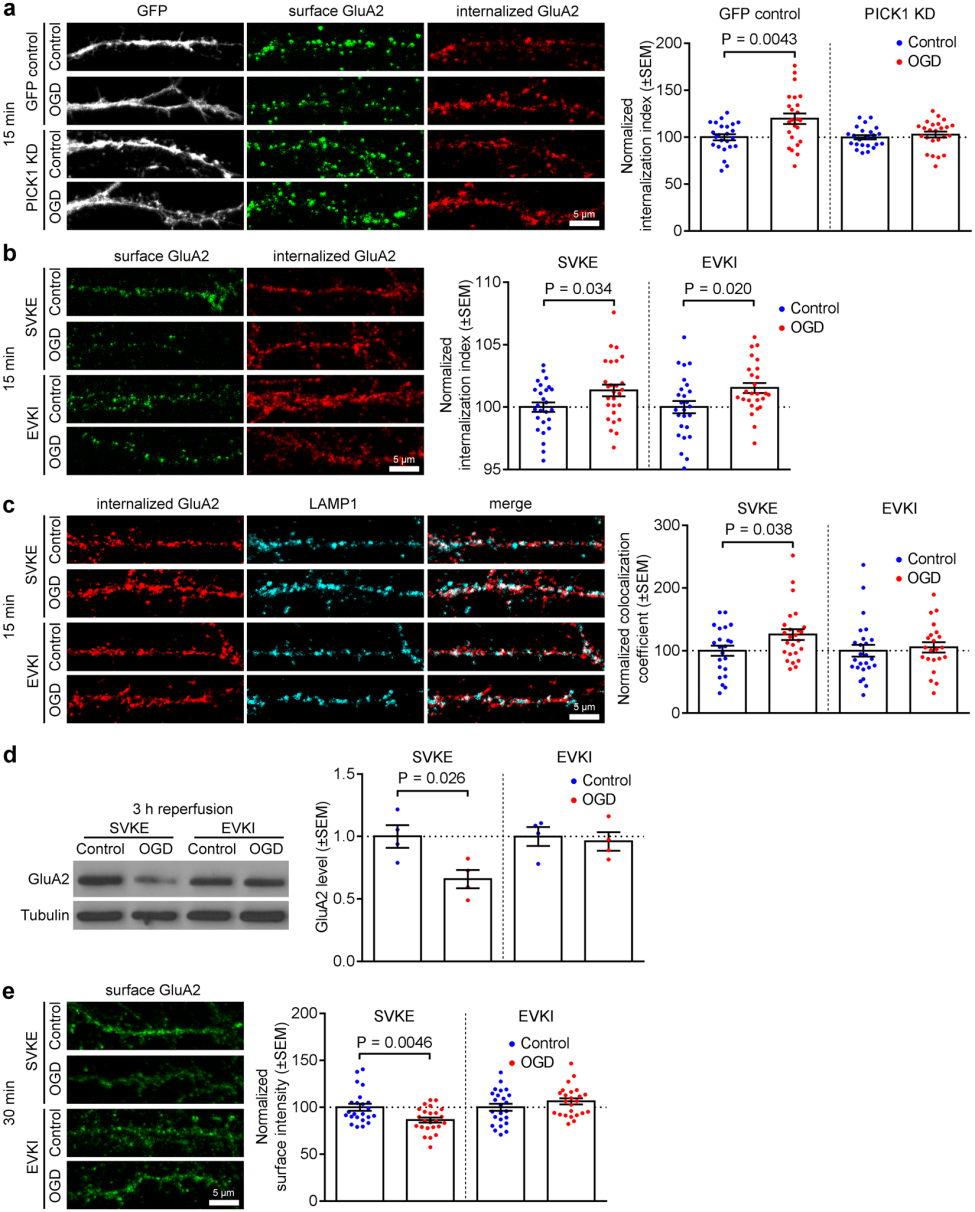
PICK1 is required for endocytosis and lysosomal targeting of endogenous GluA2 after OGD/reperfusion. (**a**) PICK1 knockdown blocks OGD-induced GluA2 internalization. Cultured hippocampal neurons were transfected with plasmids expressing PICK1 shRNA or GFP control, live-labeled with anti-GluA2 antibodies prior to 20 min OGD and 15 min reperfusion. The intensities of the remaining surface (green) and internalized (red) endogenous GluA2 were measured after fixation and used to calculate the internalization index (internalized/surface + internalized). Graph indicates that PICK1 knockdown blocked the increase in internalization index. Representative confocal images are shown. Two-tailed Student’s t-test. (**b**) TAT-EVKI does not block GluA2 endocytosis when applied during OGD. Hippocampal neurons were live-labeled with anti-GluA2 antibodies prior to 20 min OGD. TAT-EVKI (active) or TAT-SVKE (control) peptide was bath applied during OGD, followed by 15 min reperfusion in the absence of peptide. The intensities of the remaining surface (green) and internalized (red) endogenous GluA2 were measured after fixation and used to calculate the internalization index (internalized/surface + internalized), showing that TAT-EVKI does not block GluA2 endocytosis. Representative confocal images are shown. Two-tailed Student’s t-test. (**c**) TAT-EVKI blocks the colocalization between endocytosed GluA2 and LAMP1. Hippocampal neurons were treated as in b, above, followed by immunostaining for LAMP1. Pearson’s coefficients of colocalization were determined, which indicate that TAT-EVKI abolishes the increase in colocalization between iGluA2 and LAMP1 caused by OGD/reperfusion. Representative confocal images are shown. Two-tailed Student’s t-test. (**d**) TAT-EVKI blocks lysosomal degradation of GluA2 after OGD/reperfusion. Hippocampal neurons were treated with 20 min OGD, during which they were exposed to TAT-EVKI (active) and TAT-SVKE (control) peptides, followed by 3 h reperfusion. Cell lysates were analyzed by Western blotting. Graph shows total GluA2 levels. Two-tailed Student’s t-test. Blots shown are cropped; full-length blots are shown in Supplementary Fig. S3. (**e**) TAT-EVKI promotes GluA2 recycling to the cell surface after OGD/reperfusion. Hippocampal neurons were treated with 20 min OGD, during which they were exposed to TAT-EVKI (active) and TAT-SVKE (control) peptides, followed by 30 min reperfusion. Neurons were fixed and surface labeled with anti-GluA2 antibodies. TAT-EVKI peptide eliminates the decrease in surface GluA2 caused by OGD/reperfusion. Representative confocal images are shown. Two-tailed Student’s t-test.

We demonstrated previously that the PDZ and BAR domain protein PICK1 is involved in the loss of surface GluA2 caused by OGD^15^. Since PICK1 has been implicated in both endocytosis and endosomal sorting of GluA2-containing AMPARs and other membrane proteins (refs^23–29^), we asked whether it is involved in OGD-induced AMPAR endocytosis and/or lysosomal targeting. Initially we used a previously-characterized shRNA to reduce PICK1 expression in hippocampal neurons^26,30^, and found that PICK1 knockdown blocked the OGD-induced increase in GluA2 endocytosis (Fig. 3a), demonstrating that PICK1 is essential for OGD-induced endocytosis of GluA2-containing AMPARs. Since the majority of receptors that are sorted to lysosomes are originally endocytosed from the plasma membrane, this result precluded the use of shRNA to study lysosomal sorting specifically. In order to selectively block a putative function of PICK1 in AMPAR endo/lysosomal sorting, we investigated the effect of acutely disrupting PICK1 binding to GluA2 at a time point after this protein-protein interaction is required for AMPAR endocytosis. We used a peptide corresponding to the last 10 amino acids of the GluA2 C-terminus carrying a serine to glutamate substitution at the −3 position (-EVKI) to selectively disrupt GluA2-PICK1 binding. A similar peptide with an isoleucine to glutamate substitution at the 0 position (-SVKE) serves as an inactive control^31^. In a previous study, we expressed these peptides using Sindbis virus, such that they were present prior to OGD and therefore were available for blocking the endocytic function of PICK1^15^. Under those conditions, -EVKI peptide blocked the OGD-induced loss of surface GluA2. In the current study, we bath-applied cell-permeant TAT-tagged peptides, and investigated changes in GluA2 endocytosis, lysosomal targeting and surface levels in response to OGD. We aimed to time the peptide application so that intracellular levels were maximal at the time of lysosomal targeting, but insufficient at the time that PICK1-GluA2 interactions initiate endocytosis. Since it has been shown that TAT-tagged peptides take longer than 30 min to reach maximal intracellular concentration in cultured neurons^32^, we applied the peptide during and after OGD. First, we analysed GluA2 endocytosis by antibody-feeding assay, and found that there was no difference between TAT-SVKE and TAT-EVKI conditions, indicating that the acute application of TAT-EVKI peptide had no effect on OGD-induced endocytosis of GluA2-containing AMPARs (Fig. 3b). Since our results shown in Fig. 3a indicate that PICK1 is required for OGD-induced GluA2 endocytosis, we conclude that in the peptide experiments, TAT-EVKI had not reached a high enough intracellular concentration by the time PICK1-GluA2 interactions initiate endocytosis. Since lysosomal targeting of internalized receptors necessarily happens after endocytosis, we investigated whether this treatment affected lysosomal targeting by analysing iGluA2/LAMP1 colocalization following bath application of the peptides (Fig. 3c). In the presence of the control TAT-SVKE peptide, OGD caused an increase in iGluA2/LAMP1 colocalization, similar to the experiments carried out in the absence of peptide (see Fig. 2a). In contrast, TAT-EVKI completely blocked the OGD-induced increase in iGluA2/LAMP1 colocalization, suggesting that the PICK1-GluA2 interaction is involved in the targeting of GluA2-containing AMPARs to lysosomes. Furthermore, TAT-EVKI completely blocked OGD-induced lysosomal degradation of GluA2-containing AMPARs (Fig. 3d). The reduction in lysosomal targeting caused by TAT-EVKI suggested that the internalized receptors were instead recycled back to the plasma membrane under these conditions. To test this hypothesis, we carried out surface staining of GluA2 30 min after OGD. In the presence of TAT-EVKI, GluA2-containing AMPARs had returned to the surface, whereas in the presence of TAT-SVKE, OGD caused a decrease in surface GluA2 (Fig. 3e). Since TAT-EVKI did not block GluA2 endocytosis (see Fig. 3b), we conclude that this effect was caused by altered endosomal sorting. Taken together, these results suggest that PICK1 is required for the OGD-induced endocytosis of endogenous GluA2-containing AMPARs, and is also an essential component of the mechanism that underlies the lysosomal targeting of GluA2-containing AMPARs caused by OGD.

### PICK1-AP2 interaction is enhanced by OGD in hippocampal but not cortical neurons

**Figure 4:**
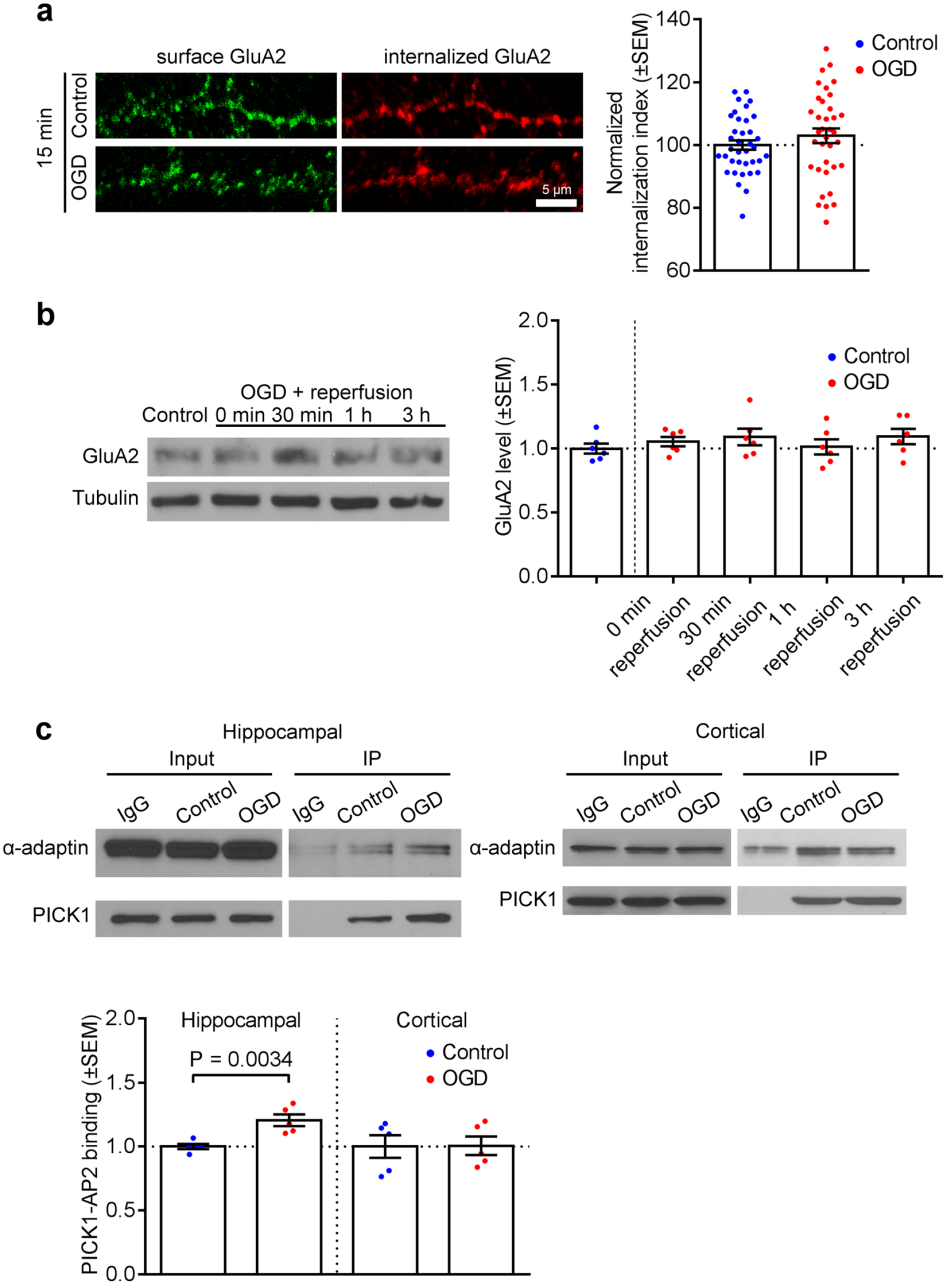
GluA2 endocytosis and PICK1-AP2 interaction are increased by OGD/reperfusion in hippocampal, but not cortical neurons. (**a**) GluA2 is not endocytosed in cortical neurons following OGD/reperfusion. Neurons were live-labeled with anti-GluA2 antibodies prior to 20 min OGD and 15 min reperfusion. The intensities of the remaining surface (green) and internalized (red) endogenous GluA2 were measured after fixation and used to calculate the internalization index (internalized/surface + internalized). Representative confocal images of dendrites from cortical cultures are shown. Two-tailed Student’s t-test. (**b**) Endogenous GluA2 is not degraded following OGD and reperfusion in cultured cortical neurons. Cell lysates were analyzed by Western blotting. Graph shows total GluA2 levels following 20 min OGD and 0, 30 min, 1 h and 3 h reperfusion. One-way ANOVA followed by Dunnett’s test. Blots shown are cropped; full-length blots are shown in Supplementary Fig. S3. (**c**) OGD causes an increase in endogenous PICK1-AP2 interactions in hippocampal but not cortical neurons. Extracts of cultured hippocampal and cortical neurons were immunoprecipitated with PICK1 antibodies or control IgG. Proteins were detected with anti-PICK1 and anti-α-adaptin (AP2) antibodies. Input is 5% of offered protein. Two-tailed Student’s t-test. Blots shown are cropped; full-length blots are shown in Supplementary Fig. S3.

We previously demonstrated that cultured hippocampal and cortical neurons exhibit distinct patterns of AMPAR trafficking, in particular the OGD-induced loss of surface GluA2 occurs in hippocampal but not cortical neurons^16^. The absence of this change in cortical neurons could be explained by a rapid recycling of GluA2 back to the cell surface following OGD-induced endocytosis, or alternatively by an absence of any increase in GluA2 endocytosis. To investigate this, we used antibody-feeding assays on cortical neurons to assess GluA2 endocytosis during OGD. In contrast to hippocampal neurons (see Fig. 1c), GluA2 endocytosis and degradation were unaffected by OGD in cortical neurons (Fig. 4a,b). Since our results indicate that PICK1 is a critical component of OGD-induced GluA2 endocytosis, and we have recently demonstrated that PICK1 interacts with the endocytic adaptor complex AP2^28^, we asked whether the interaction between PICK1 and AP2 can be regulated by OGD to mediate AMPAR endocytosis. We carried out co-immunoprecipitations from neuronal lysates immediately after OGD, which showed that OGD caused an increase in the association of PICK1 with AP2 in hippocampal, but not cortical neurons (Fig. 4c). This suggests intrinsic differences in the molecular cell biology of cortical and hippocampal neurons, manifested by a differential effect of OGD on the PICK1-AP2 interaction.

### GluA3, but not GluA1, follows the same OGD-induced trafficking as GluA2 in hippocampal neurons

**Figure 5:**
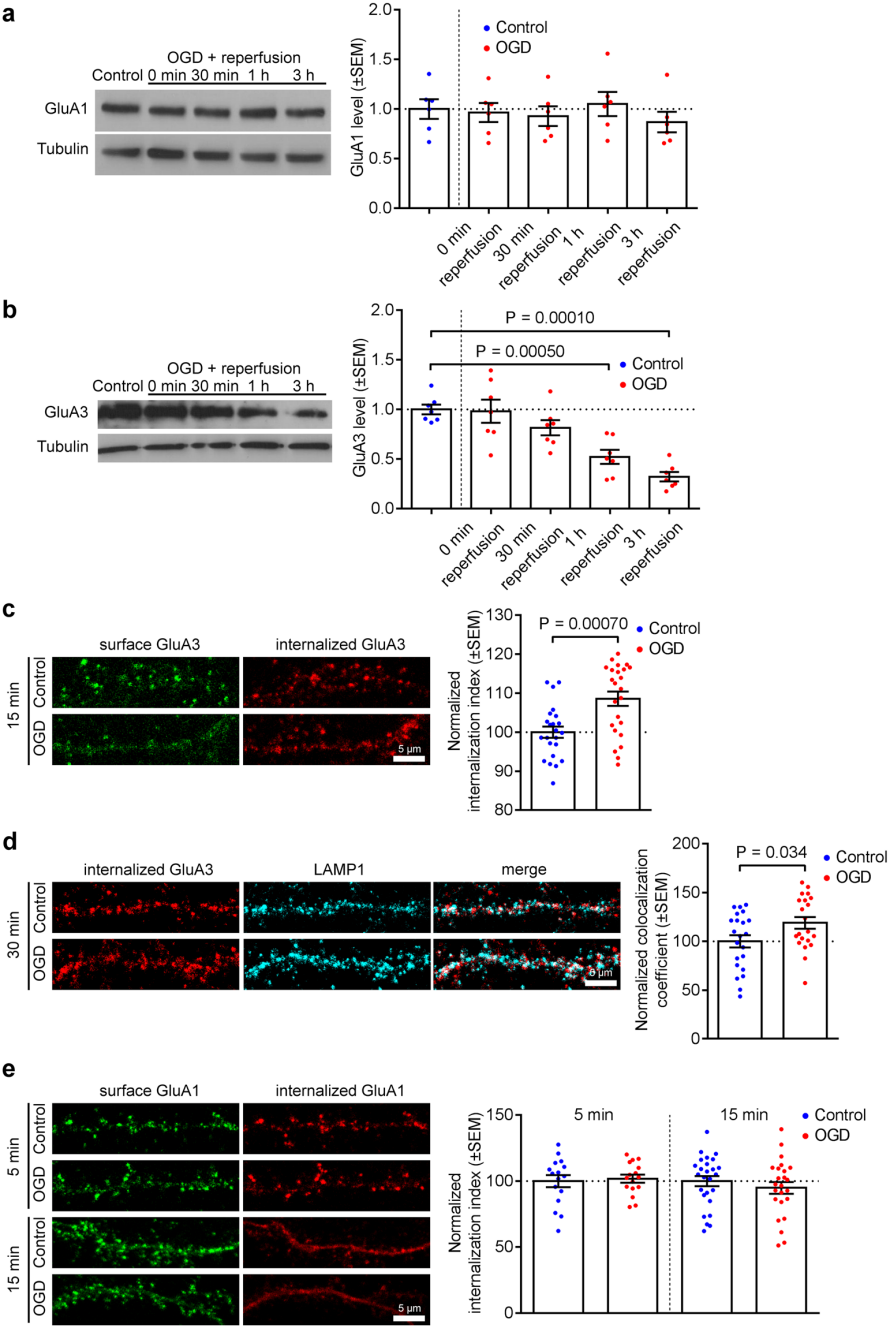
GluA3, but not GluA1, is endocytosed and degraded in response to OGD in hippocampal neurons. (**a**) GluA1 is not degraded following OGD/reperfusion in cultured hippocampal neurons. Cell lysates were analyzed by Western blotting. Graph shows total GluA1 levels following 20 min OGD and 0, 30 min, 1 h and 3 h reperfusion. One-way ANOVA followed by Dunnett’s test. Blots shown are cropped; full-length blots are shown in Supplementary Fig. S3. (**b**) GluA3 is degraded following OGD/reperfusion in hippocampal neurons. Cell lysates were analyzed by Western blotting. Graph shows total GluA3 levels following 20 min OGD and 0, 30 min, 1 h and 3 h reperfusion. One-way ANOVA followed by Dunnett’s test. Blots shown are cropped; full-length blots are shown in Supplementary Fig. S3. (**c**) OGD/reperfusion causes endocytosis of endogenous GluA3 in hippocampal neurons. Neurons were live-labeled with anti-GluA3 antibodies prior to 20 min OGD and 15 min reperfusion. The intensities of the remaining surface (green) and internalized (red) endogenous GluA3 were measured after fixation and used to calculate the internalization index (internalized/surface + internalized). Representative confocal images of dendrites are shown. Two-tailed Student’s t-test. (**d**) Lysosomal targeting of internalized endogenous GluA3 following OGD in hippocampal neurons. OGD/reperfusion causes the targeting of internalized endogenous GluA3 to lysosomes in dendrites of cultured hippocampal neurons. Neurons were live-labeled with anti-GluA3 antibodies prior to 20 min OGD and 30 min reperfusion. After fixation, internalized GluA3 was labeled with red secondary antibodies, and neurons were also immunostained for the lysosomal marker LAMP1 (cyan). Representative confocal images are shown. Pearson’s coefficient of colocalization between internalized GluA3 and the lysosomal marker LAMP1 was determined. Graph indicates a significant increase after 30 min reperfusion. Two-tailed Student’s t-test. (**e**) OGD/reperfusion does not cause endocytosis of endogenous GluA1 in hippocampal neurons. Neurons were live-labeled with anti-GluA1 antibodies prior to 20 min OGD and 5 and 15 min reperfusion. The intensities of the remaining surface (green) and internalized (red) endogenous GluA1 were measured after fixation and used to calculate the internalization index (internalized/surface + internalized). Representative confocal images of dendrites are shown. Two-tailed Student’s t-test.

Since GluA2 homomers are thought not to exist *in vivo* ^6^, a critical question is whether the GluA2-dependent processes defined in our experiments represent trafficking of GluA1/2 or GluA2/3 complexes. A previous report suggested that both heteromeric assemblies were internalized in response to OGD, and GluA1/3 receptors were subsequently inserted at the plasma membrane^14^. To investigate which subunits undergo OGD-induced degradation, we analysed total levels of GluA1 and GluA3 in hippocampal neurons after insult by Western blotting. OGD had no effect on GluA1 subunit (Fig. 5a), but caused a significant decrease in GluA3 levels (Fig. 5b), which followed a similar pattern of degradation as GluA2 (see Fig. 2c). This suggested that GluA2/3, but not GluA1/2 receptors were targeted for lysosomal degradation. To investigate at which point in the endocytic/endosomal pathway this specificity was determined, we carried out antibody-feeding assays to analyse OGD-induced endocytosis of endogenous GluA1 or GluA3 in hippocampal neurons. Interestingly, GluA3 showed a similar pattern of trafficking events as GluA2, with a robust increase in endocytosis at 15 min after OGD compared to controls (Fig. 5c). Furthermore, colocalization between iGluA3 and LAMP1 was significantly increased at 30 min after OGD (Fig. 5d). In contrast, we were unable to detect any effect of OGD on GluA1 endocytosis (Fig. 5e). To investigate the possibility that GluA1 endocytosis was stimulated rapidly during OGD, and internalized receptors had already recycled to the plasma membrane by 15 min post-OGD, we also quantified endocytosed GluA1 at 5 min after OGD. Even at this earlier time point, there was no OGD-induced increase in GluA1 internalization (Fig. 5e). These results strongly suggest that the endogenous AMPAR complexes endocytosed and subsequently degraded are GluA2/3 heteromers. Surprisingly, GluA1/2 heteromeric receptors appear to be excluded from GluA2-driven endocytosis in response to OGD.

## Discussion

Our results indicate that OGD causes a selective PICK1-dependent endocytosis of endogenous AMPAR subunits GluA2 and GluA3 in hippocampal neurons, but not in cortical neurons, which can at least partly be explained by a cell-type specific OGD-induced increase in PICK1 binding to the endocytic adaptor complex AP2. The internalized GluA2/3 receptors are targeted to lysosomes, also in a PICK1-dependent manner, where they are degraded. GluA1-containing AMPARs are not internalized in response to OGD, suggesting a mechanism that protects surface GluA1 from endocytosis.

It has been known for some time that GluA2 subunit expression is reduced in hippocampal CA1 neurons following ischaemia, which has until now been fully attributed to transcriptional down-regulation of GluA2 mRNA^17,18^. Our results demonstrate that a more rapid OGD-induced down-regulation of total GluA2 is caused by lysosomal targeting of AMPARs, indicating that reduced GluA2 protein expression is a two-step process; lysosomal degradation of GluA2 protein is detectable 1 h after OGD, while a reduction in GluA2 mRNA is detectable by 24 h after OGD or ischaemia^18,33^. The critical outcome of these changes is a reduction in GluA2 content at the synaptic plasma membrane, and consequent expression of CP-AMPARs that contribute to OGD-induced neuronal death^15^. Our results suggest that, while inhibition of lysosomal degradation *per se* does not promote GluA2 recycling, acute blockade of GluA2 targeting to lysosomes by TAT-EVKI redirects GluA2 back to the cell surface. This suggests that the PICK1-dependent endo-lysosomal sorting event is critical to the rapid loss of surface GluA2, even prior to its degradation.

A previous model proposed to define subunit-specific trafficking mechanisms that underlie the cell-surface expression of CP-AMPARs suggests that GluA1, GluA2 and GluA3 are endocytosed in response to OGD, followed by the plasma membrane insertion of GluA1 and GluA3 while GluA2 is retained in an intracellular compartment^14^. In this model, the endocytosed GluA1 is precisely balanced by the exocytosed GluA1 in spite of the fact that at least a proportion of the GluA1 inserted at the plasma membrane (as GluA1/3 heteromers or GluA1 homomers) must be a different pool of receptors to those internalized and retained inside the cell (as GluA2-containing heteromers). In striking contrast, our results indicate that GluA2 and GluA3 are specifically endocytosed, while GluA1 is protected from endocytosis by an unknown mechanism. The difference in conclusions between our work and the previous study may be explained by differences in methodology to analyse GluA1 internalization. Liu *et al*. used hypertonic sucrose to globally inhibit endocytosis, revealing plasma membrane insertion of GluA1 in response to OGD^14^. In contrast, we directly tested GluA1 internalization using antibody-feeding assays. The absence of any endogenous GluA1 endocytosis in response to OGD is surprising, since PICK1 would be expected to influence endocytosis of GluA1/2 heteromers. How GluA1-containing receptors resist endocytosis after OGD is unclear. Hippocampal NMDAR-dependent LTD is PICK1-dependent, and also involves endocytosis of GluA1-containing AMPARs^34,35^, hence our results highlight a mechanistic difference in the trafficking events associated with OGD and LTD, and suggest a GluA1-dependent process is specifically recruited by OGD to retain GluA1-containing AMPARs at the cell surface. If GluA1 is neither internalized nor exocytosed, the synaptic incorporation of CP-AMPARs must involve the lateral movement of GluA1/3 or GluA1 homomers from extrasynaptic sites, which has been suggested previously in other models for the synaptic recruitment of CP-AMPARs, for example as a critical component of LTP expression^36^.

We previously demonstrated that OGD increased the endocytosis of overexpressed GluA2 homomers in hippocampal but not cortical neurons^16^. Consistent with this observation, and in a critical advance on these previous experiments, we show here that endogenous GluA2-containing AMPARs are endocytosed in response to OGD in hippocampal, but not cortical neurons. Furthermore, we show that the interaction between PICK1 and the endocytic adaptor complex AP2 is enhanced by OGD in hippocampal but not cortical neurons, indicating that this intrinsic component of molecular cell biology is different between the two cell types. Interestingly, we find that the PICK1-AP2 interaction in cortical neurons is increased by bath application of NMDA^28^, indicating a further difference in the neuronal response to OGD compared to chemically-induced LTD. We previously demonstrated that disrupting PICK1-GluA2 interactions (albeit chronically) in hippocampal neurons protects hippocampal neurons from OGD-induced neuronal death^15^, supporting the conclusion that PICK1 plays a key role in the vulnerability of hippocampal neurons to OGD. The absence of PICK1-dependent GluA2-containing AMPAR endocytosis, and the consequent absence of GluA2 lysosomal degradation following OGD in cortical neurons suggests that OGD does not cause CP-AMPAR expression in cortical neurons. We therefore propose that this difference in trafficking contributes to the lesser vulnerability to ischaemic insult of cortical neurons compared to hippocampals.

PICK1 has previously been implicated in restricting GluA2 recycling by retaining internalized receptors in the endosomal system, leading to reduced synaptic receptors during LTD expression^24,26,27^. Our results suggest that PICK1 plays a role in targeting GluA2 to lysosomes, and that an acute and timely inhibition of PICK1 function redirects GluA2-containing AMPARs away from a lysosomal destination, and causes their recycling to the plasma membrane. Hence our results are consistent with previous studies suggesting an endosomal sorting role for PICK1, yet give further mechanistic insight into PICK1 function. Our results also suggest that PICK1 plays a role in both endocytosis and in endo/lysosomal sorting in response to the same stimulus. The precise mechanisms and signalling pathways upstream of PICK1 that determine these distinct trafficking events are unknown at this time, but warrant further investigation.

Our work suggests that the relatively late event of GluA2 lysosomal targeting could be a target for therapeutic intervention. Acute application of a peptide or other compound to block GluA2 degradation and redirect GluA2 back to the synaptic plasma membrane might prevent the accumulation of CP-AMPARs at synaptic sites, and hence reduce neuronal death following global cerebral ischaemia.

## Methods

### Antibodies

The antibodies used were as follows: α-adaptin (Becton Dickinson); GluA1 (Millipore); GluA2 (rabbit Synaptic Systems, mouse Becton Dickinson); GluA3 (Alomone); LAMP1 (Abcam); PICK1 (rabbit Abcam, mouse Neuromab); tubulin (Sigma); Rab5 (Cell Signaling); Rab11 (Cell Signaling).

### Co-immunoprecipitation

Extracts of hippocampal and cortical neuronal cultures were prepared in lysis buffer (50 mM Tris pH 7.4, 150 mM NaCl, 0.5% Triton X-100, plus Roche protease inhibitor cocktail) and subsequently incubated with 5 µg anti-PICK1 or control IgG antibodies followed by protein-G sepharose beads (GE Healthcare). Beads were then washed and bound proteins detected by Western blotting with anti-PICK1 and anti-α-adaptin antibodies. Western blotting films were scanned and band intensities were quantified using NIH ImageJ Fiji software. For quantification, AP2 intensity values were normalized to the immunoprecipitated PICK1 values.

### Quantification of AMPAR subunit levels by Western blotting

Hippocampal and cortical neurons were lysed immediately or 30 min, 1 h, or 3 h following OGD (50 mM Tris pH 7.8, 150 mM NaCl, 0.1% SDS, 0.5% sodium deoxycholate, 1% Triton X-100, and Roche protease inhibitor cocktail). Western blotting films were scanned and band intensities were quantified using NIH ImageJ Fiji software. For quantification, values were normalized to the tubulin loading control and subsequently normalized to the control condition.

### Primary neuronal culture

All animal handling procedures were approved by the Animal Welfare and Ethical Review Body of the University of Bristol, and were conducted in accordance with the UK Animals (Scientific Procedures) Act 1986. Rat embryonic neuronal cultures were prepared from E18 Wistar rats using standard procedures. The culture medium was Neurobasal medium (Gibco) supplemented with B27 (Gibco) and 2 mM glutamine. Primary neuronal cultures were used for experiments at DIV 14-20 days. For PICK1 KD experiments, neurons were transfected with plasmid DNA at DIV 9–13 using Lipofectamine 2000 (Invitrogen), and used for experiments 5 days later. PICK1 shRNA and control constructs were expressed from a modified FUGW vector (Antoniou *et al*.^30^).

In order to block lysosomal degradation following OGD, 100 μg/ml leupeptin was added to the culture medium for 3 h before and during OGD/reperfusion. To block PICK1-GluA2 interactions, 10 µM cell-permeable TAT-EVKI and control SVKE peptides (custom-made by GenScript, USA) were bath-applied during OGD.

### Oxygen/Glucose Deprivation

Neuronal cultures were washed three times with HEPES buffered saline (25 mM HEPES pH 7.4, 137 mM NaCl, 5 mM KCl, 1.5 mM CaCl_2_, 1.5 mM MgCl_2_, 15 mM sucrose for OGD condition or 15 mM glucose for control condition) and incubated in a hypoxic chamber (MACS-VA500-microaerophilic workstation; Don Whitley Scientific, West Yorkshire, UK) for 20 min at 37 °C, 95% N2 and 5% CO2. For OGD condition, buffer was previously deoxygenated overnight in the hypoxic chamber. Control cultures were incubated under normoxic conditions at 37 °C, 5% CO_2_ for the same time period with HEPES buffered saline, containing glucose.

### Immunocytochemistry and image analysis

For surface staining of AMPARs, neurons were fixed under non-permeabilizing conditions with 4% PFA + 4% sucrose for 5 min. Neurons were then surface labelled with anti-GluA2 antibodies for 1 h at RT, followed by anti-mouse Alexa 568 secondary antibodies.

For antibody feeding experiments, live hippocampal or cortical neurons were surface treated with anti-GluA1/2/3 antibodies for 15 min at 20 °C, followed by 20 min of OGD and a range of reperfusion times. Neurons were fixed under non-permeabilizing conditions with 4% PFA + 4% sucrose for 5 min, followed by labelling of surface AMPA receptors with anti-mouse Alexa 488. Neurons were then fixed again for 5 min with 4% PFA + 4% sucrose and permeabilized with 0.2% Triton X-100. Validation of the antibody feeding assay was performed by omitting the permeabilization step and by using 300 mM sucrose to block the endocytosis of surface labelled receptors (Supplementary Fig. S4).

Anti-LAMP1 primary antibodies were added for 1 h at RT, followed by anti-mouse Alexa 647 to visualize internalized GluA1/2/3 receptors and anti-rabbit Alexa 568 to visualize LAMP1.

Images were acquired on a Leica SP8 confocal microscope with a 63x objective (NA 1.4) and analyzed using NIH ImageJ, Fiji software. Three randomly selected ~30 µm dendritic regions were used per neuron and values were averaged. Internalization index was calculated by dividing the intensity corresponding to the internalized staining by the values corresponding to the total staining (internalized + surface). OGD treated groups were normalized to the corresponding control groups. Pearson’s colocalization analysis was performed using the Coloc 2 plugin in ImageJ Fiji. Pearson’s colocalization coefficients in OGD treated groups were normalized to the control groups within each dissection, to avoid the variation among different neuronal dissections. Raw Pearson values can be found in Supplementary Table S1. Line scans showing colocalization between internalized GluA2 and LAMP1 can be found in Supplementary Fig. S5.

### Statistical analysis

All statistical analyses were performed by using GraphPad Prism version 7.00 (GraphPad Software, USA). Data are presented as mean ± SEM. Scatter plots represent the number of neurons, which were analyzed from 3 independent experiments taken from different dissections.

OGD treated internalization indices and colocalization coefficients were normalized to the control conditions separately for each dissection/set of experiment. Normalization was necessary to account for small, yet inevitable differences between experiments carried out on cultures from different neuronal dissections. Each set of experiments included both control and OGD groups. The mean value of the control group was considered 100% and OGD treated groups were normalized accordingly. The magnitude of change between control and OGD conditions for each dissection/set of experiment, the N number, and variance of the data were maintained. Raw Pearson values can be found in Supplementary Table S1.

In the case of Western blotting, scatter plots represent the number of culture dishes used, also taken from different dissections. Statistical significance was accepted at P < 0.05. Normality of the data was tested using the D’Agostino-Pearson normality test.

## Electronic supplementary material

Supplementary Information
