# Supplementary material for: Endocytosis and lysosomal degradation of GluA2/3 AMPARs in response to oxygen/glucose deprivation in hippocampal but not cortical neurons
Source: Sci Rep. 2017 Sep 26;7:12318. doi: 10.1038/s41598-017-12534-w (PMC5614987; doi:10.1038/s41598-017-12534-w)
Supplement: Supplementary file 1 — Supplementary Information [file 41598_2017_12534_MOESM1_ESM.pdf]

Endocytosis and lysosomal degradation of GluA2/3 AMPARs in response to oxygen/glucose deprivation in hippocampal but not cortical neurons

Zsombor Koszegi, Maria Fiuza, Jonathan G Hanley

Supplementary Figure S1

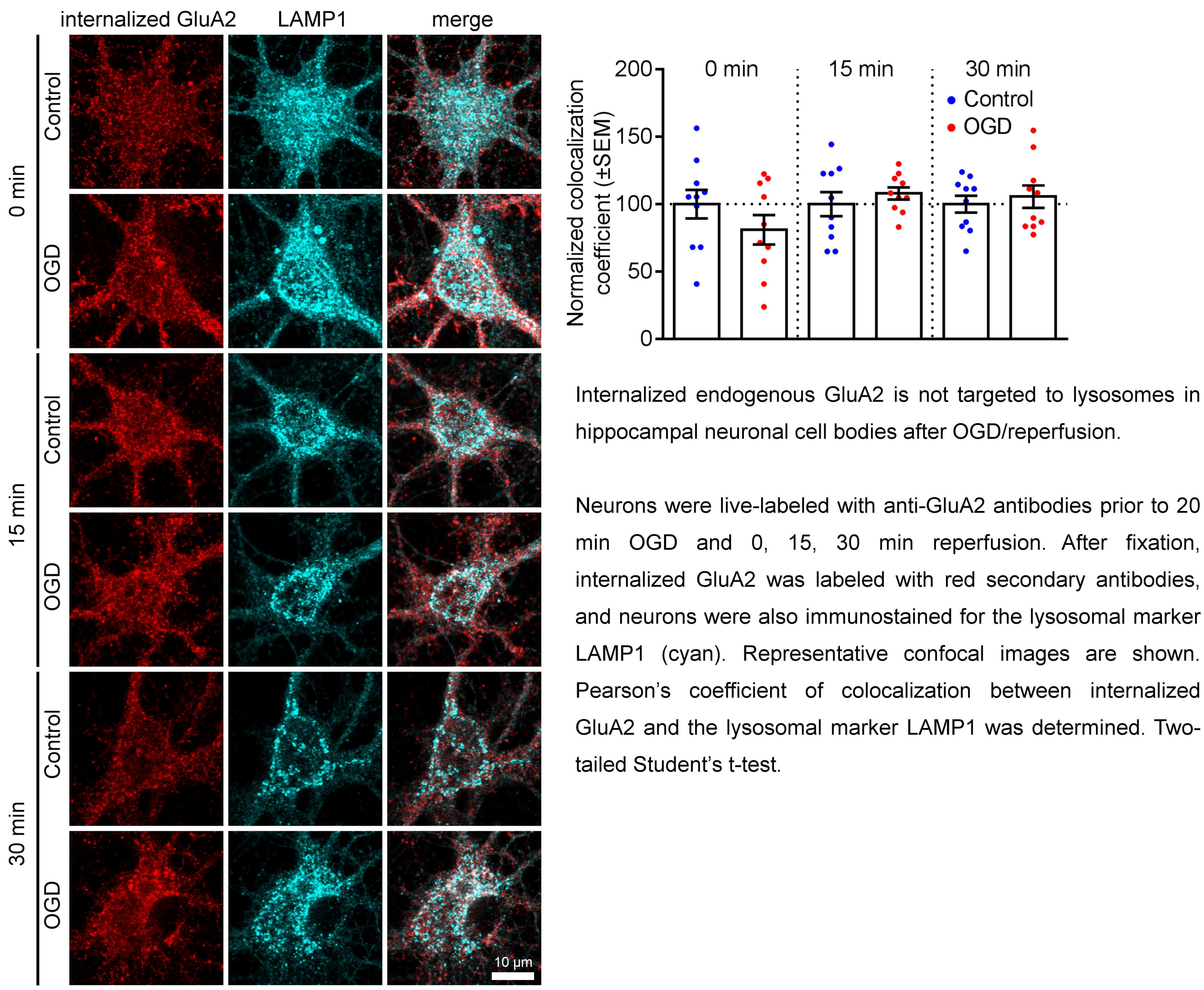

Internalized endogenous GluA2 is not targeted to lysosomes in hippocampal neuronal cell bodies after OGD/reperfusion.

Neurons were live-labeled with anti-GluA2 antibodies prior to 20 min OGD and 0, 15, 30 min reperfusion. After fixation, internalized GluA2 was labeled with red secondary antibodies, and neurons were also immunostained for the lysosomal marker LAMP1 (cyan). Representative confocal images are shown. Pearson's coefficient of colocalization between internalized GluA2 and the lysosomal marker LAMP1 was determined. Two-tailed Student's t-test.

Supplementary Figure S2

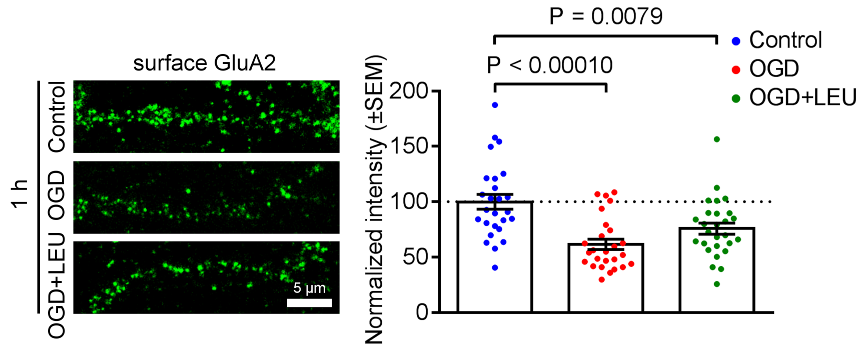

The loss of surface GluA2 caused by OGD is unaffected by leupeptin treatment.

Neurons were treated with leupeptin for 3 hours then live-labeled with anti-GluA2 antibodies prior to 20 min OGD and 1 h reperfusion. After fixation, surface GluA2 was labeled with green antibodies. Representative confocal images are shown. One-way ANOVA followed by Tukey’s test.

Supplementary Figure S3

Figure 2c

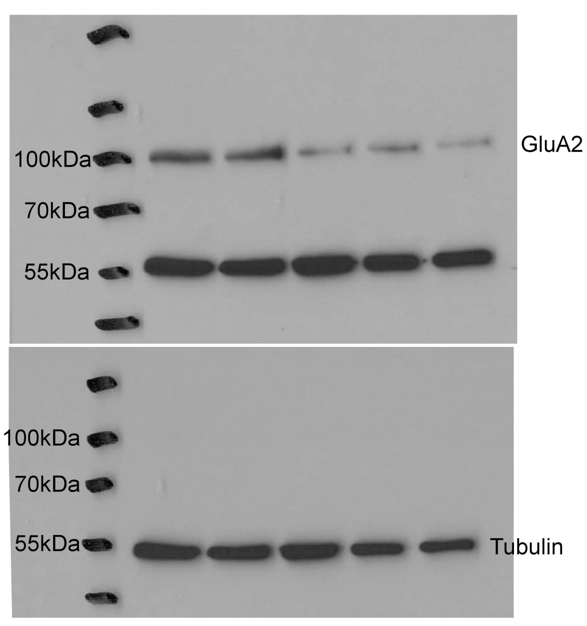

Figure 2d

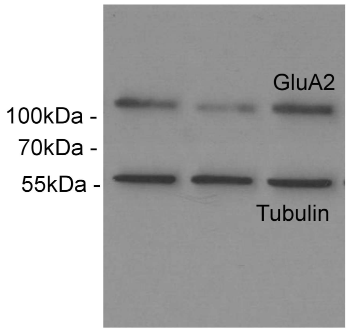

Figure 3d

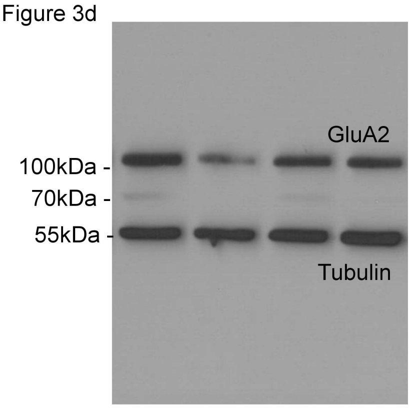

Figure 4b

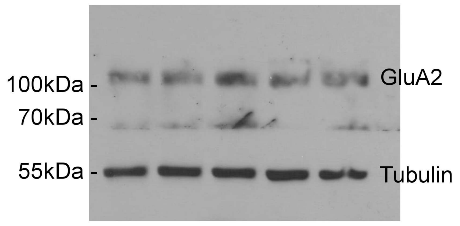

Figure 4c

Hippocampal

Cortical

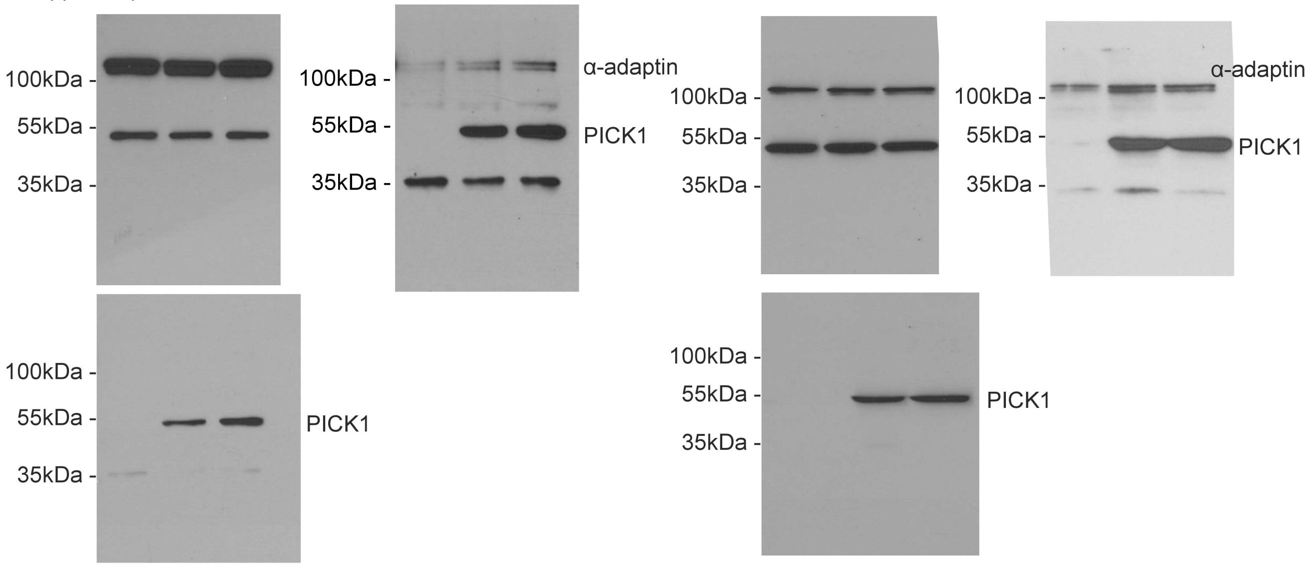

Figure 5a

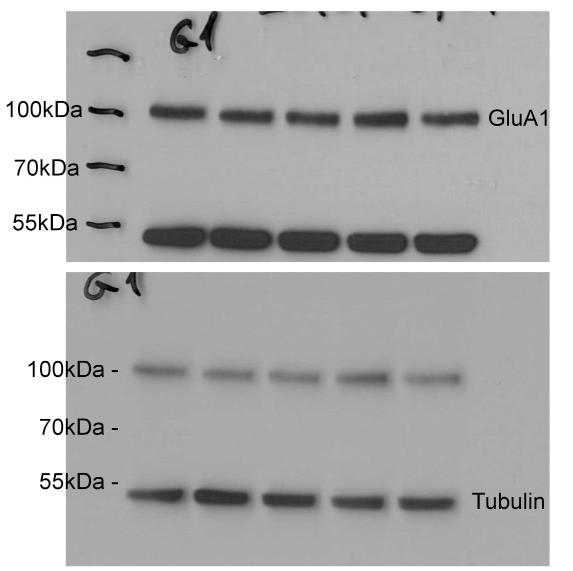

Figure 5b

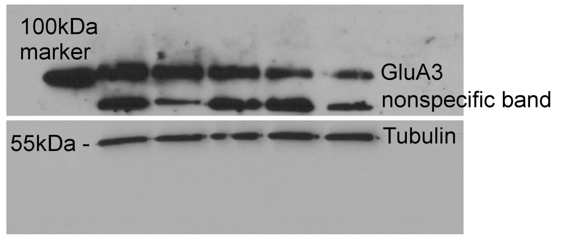

Supplementary Figure S4

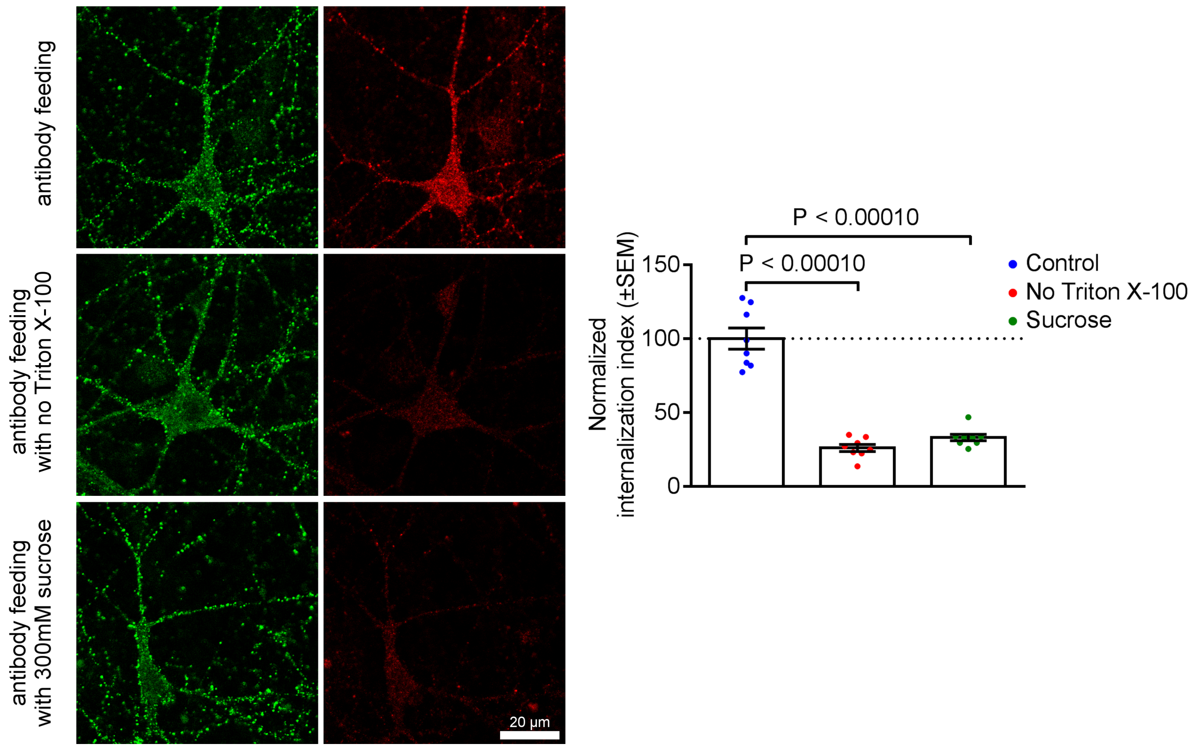

Negative controls demonstrate the selective labeling of internalized GluA2 receptors.

Internalization index was calculated following GluA2 antibody feeding. Omitting a permeabilization step (no Triton X-100) results in a significant decrease in the internalized GluA2 intensity. Similarly, blocking endocytosis with 300mM sucrose results in a significant decrease in the internalized GluA2 intensity. One-way ANOVA followed by Tukey's test.

Supplementary Figure S5

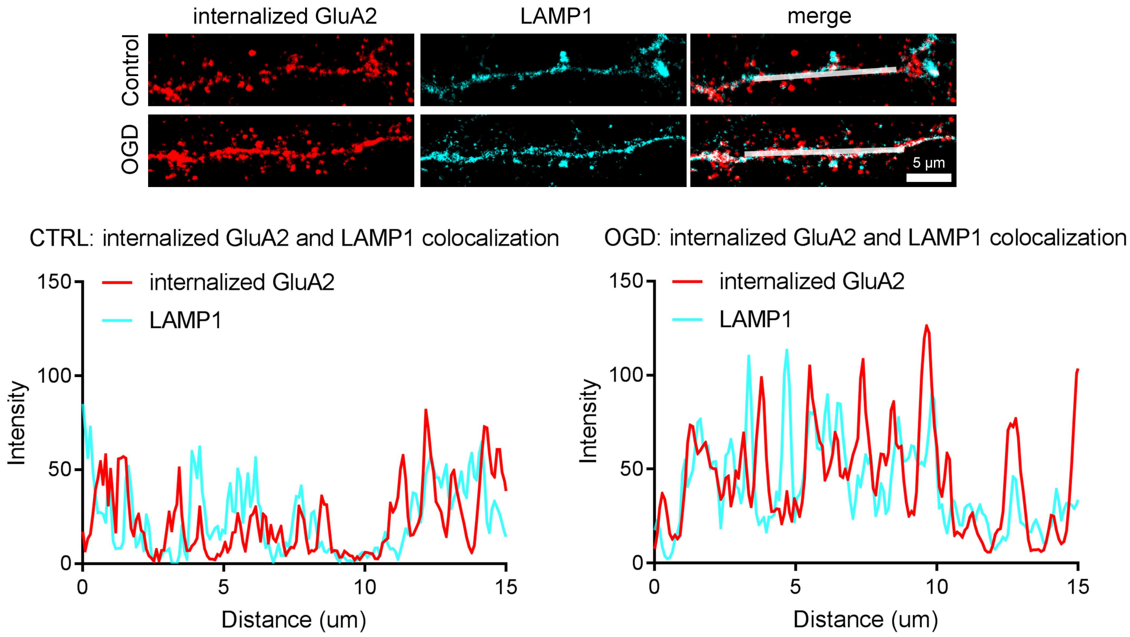

Internalized endogenous GluA2 colocalizes with LAMP1 following OGD/reperfusion in hippocampal neurons.

Line scans indicate the level of colocalization after 20 min of OGD (or control), followed by 30 min of reperfusion.

Supplementary Table S1

		mean	SD
**Figure 2a**			
0 min	CTRL	0.3942	0.0843
	OGD	0.3943	0.0920
15 min	CTRL	0.3921	0.0869
	OGD	0.4264	0.0952
30 min	CTRL	0.4471	0.0387
	OGD	0.4975	0.0864
**Figure 2b**			
1 h	CTRL	0.3035	0.0452
	OGD	0.3375	0.0918
	OGD+LEU	0.3771	0.0837
**Figure 2e**			
Rab5			
15 min	CTRL	0.1865	0.0508
	OGD	0.2253	0.0371
Rab11			
15 min	CTRL	0.3156	0.0549
	OGD	0.3141	0.0549
**Figure 3c**			
SVKE			
15 min	CTRL	0.1489	0.0694
	OGD	0.1947	0.0885
EVKI			
15 min	CTRL	0.1728	0.0870
	OGD	0.1891	0.0772
**Figure 5d**			
30 min	CTRL	0.1455	0.0425
	OGD	0.1746	0.0381

Table showing the raw values for Pearson’s coefficient of colocalization.
